# Measurement-based preparation of stable coherent states of a Kerr parametric oscillator

**DOI:** 10.1038/s41598-023-28682-1

**Published:** 2023-01-28

**Authors:** Yuta Suzuki, Shohei Watabe, Shiro Kawabata, Shumpei Masuda

**Affiliations:** 1 Department of Physics, Faculty of Science Division I, Tokyo University of Science, 1-3 Kagurazaka, Shinjuku-ku, Tokyo 162-8601 Japan; 2 Research Center for Emerging Computing Technologies (RCECT), National Institute of Advanced Industrial Science and Technology (AIST), 1-1-1, Umezono, Tsukuba, Ibaraki 305-8568 Japan; 3 Department of Computer Science and Engineering, College of Engineering, Shibaura Institute of Technology, 3-7-5 Toyosu, Koto-ku, Tokyo 135-8548 Japan; 4 NEC-AIST Quantum Technology Cooperative Research Laboratory, National Institute of Advanced Industrial Science and Technology (AIST), Tsukuba, Ibaraki 305-8568 Japan

**Keywords:** Qubits, Theoretical physics

## Abstract

Kerr parametric oscillators (KPOs) have attracted increasing attention in terms of their application to quantum information processing and quantum simulations. The state preparation and measurement of KPOs are typical requirements when used as qubits. The methods previously proposed for state preparations of KPOs utilize modulation of external fields such as a pump and drive fields. We study the stochastic state preparation of stable coherent states of a KPO with homodyne detection, which does not require modulation of external fields, and thus can reduce experimental efforts and exclude unwanted effects of possible imperfection in control of external fields. We quantitatively show that the detection data, if averaged over an optimal averaging time to decrease the effect of measurement noise, has a strong correlation with the state of the KPO, and therefore can be used to estimate the state (stochastic state preparation). We examine the success probability of the state estimation taking into account the measurement noise and bit flips. Moreover, the proper range of the averaging time to realize a high success probability is obtained by developing a binomial-coherent-state model, which describes the stochastic dynamics of the KPO under homodyne detection.

## Introduction

Kerr parametric oscillators (KPOs)^1–3^ or Kerr-cat qubits, which are parametric phase-locked oscillators in the single-photon Kerr regime^4^, have attracted much attention in terms of their application to quantum information processing^5^ and study of quantum many-body systems^6,7^. KPOs can be implemented^5,8–10^ by a superconducting resonator with Kerr-nonlinearity driven by an oscillating pump field in the circuit-QED architecture. Two coherent states in opposite phases are long-lived in a KPO and their lifetime rapidly increases with the amplitude of the pump field. The long-lived coherent states, which we refer to as stable coherent states, can be used as qubit states. In the KPO, the phase-flip error dominates the bit-flip error because of the robustness of the coherent states against photon loss. Because of such a biased feature of errors, it is expected that quantum error correction for KPOs can be performed with less overhead than for qubits without such biased noise^11,12^.

Quantum annealing^3,13–19^ and universal quantum computation^3,13,20^ using KPOs were studied theoretically, and single-qubit operations were demonstrated experimentally^10^. Two-qubit gates preserving the biased feature of errors were proposed^21^, and high error-correction performance by concatenating the XZZX surface code^12^ with KPOs^22^ was numerically presented. Other research subjects on KPOs include fast gate operations and controls^9,23–26^, spectroscopy^27,28^, tomography^9,10,29^, Boltzmann sampling^30^, effects of strong pump field^31^, quantum phase transitions^6,7^, quantum chaos^1,32^, and trajectories^33–35^.

The state preparation and measurement of qubits discussed in this paper are typical requirements for developing quantum computers. For example, accurate state preparation is desirable for precise evaluation of properties of qubits such as lifetime and the fidelity of qubit gates. Importantly, the stationary state of a KPO under a pump field is a mixed state of the long-lived coherent states in contrast to transmons where a vacuum state is naturally realized as a stationary state. This is the reason why one may consider that temporal controls of system parameters are required for preparation of a pure state of a KPO. In KPO systems, preparations of predetermined qubit states were studied, using modulation of a pump field^3,13,31^ and an additional drive field^28^, of which amplitude should be surpressed over a proper period of time. However, the accuracy of the preparation method using modulation of a pump field is degraded by the decoherence caused by photon loss^31^. Although the method with an additional drive field with optimized phase and time dependent amplitude can have high accuracy, seeking such proper parameters will actually require experimental efforts, e.g., phase matching between the drive and pump fields^28^ and evaluation of the accuracy of the preparation with measurement of a KPO. In addition, the achievable preparation accuracy will be influenced by the measurement accuracy when the former is optimized using measurement results.

In this paper, we propose an alternative approach: a spontaneous and stochastic preparation of stable coherent states of KPOs based on homodyne detection, which does not require modulation of the pump field nor a drive field in contrast to the conventional methods and thus can reduce experimental efforts, and moreover we show a proper range of the averaging time to realize high success probability of the preparation. The stable coherent states are natural initial states for measurement of lifetime of a KPO, which is an essential parameter chracterizing the qubit. Therefore, it will be beneficial that a method to prepare the states is simple and independent of controllability of external fields. Very recently after the initial submission of our paper, measurement of the lifetime of the stable coherent states and their superpositions (cat states) were reported^36^. The authors prepare a stable coherent state using a measurement procedure, while their system has an auxiliary readout resonator compared to ours. Such simple measurements of the lifetime of the stable coherent state use only a continuous measurement of a KPO, and do not need repeated resets of the KPO using a temporally controlled drive field. Thus, our method can offer a simple way to extract the lifetime of a KPO. The state preparation protocol was used also to observe resonant cancellation of tunneling in a KPO^37^.

Previously, it was shown that a KPO under homodyne detection is basically in either of two coherent states with opposite phases^34,35^, and that the state of a qubit based on a KPO can be measured with homodyne detection, however without crucial analysis on detection data with measurement noise. We quantitatively show that the detection data, if it is averaged over an optimal averaging time to decrease the effect of measurement noise, has a strong correlation with the state of the KPO, and therefore can be used to accurately estimate in which coherent state the KPO is (stochastic state preparation). The success probability of the estimation (preparation) is examined taking into account the effect of the measurement noise and bit flips. We obtain the proper range of the averaging time to realize high success probability by using a developed minimum model, which describes the stochastic dynamics of the KPO under homodyne detection (similar techniques were used, e.g., to estimate the state of a two-level system^38^ and also to generate entanglement between qubits^39,40^). Moreover, we examine the dependence of the success probability on the measurement efficiency and the relative phase between the pump field and a local oscillator.

It is known that Rx^41^ and ZZ gates^42^ can be performed without modulation of the pump and drive fields. Our method of state preparation will be useful also in experimental studies of the gate operations, for example aiming at higher fidelity, because the method can exclude unwanted effects of possible imperfection in controls of the pump and drive fields. Furthermore, our method can offer implementation of the quantum information processing based on KPOs without temporal controls of the pump amplitude, because the universal gate sets^41^ can also be performed without modulation of the pump amplitude.

## Model and methods

**Figure 1:**
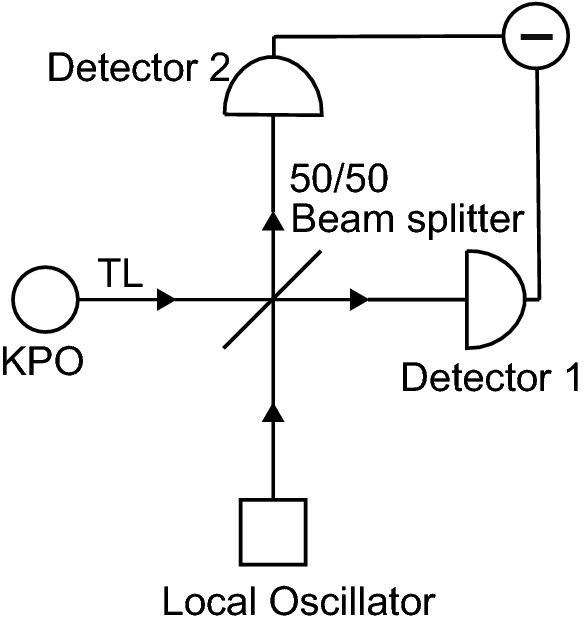
Schematic illustration of homodyne detection of a KPO attached to a transmission line (TL). The signal from the KPO and the classical coherent light from a local oscillator passing through a beam splitter are detected by detector 1 and 2. Information about the KPO is obtained after subtraction of the photocurrents at the detector 1 and 2 (Circle with a horizontal line).

We consider homodyne detection of a KPO illustrated in Fig. 1. Classical coherent light generated by a local oscillator and microwave photons emitted from the KPO are splitted by a 50/50 beam splitter and are detected at detectors 1 and 2. The KPO is connected to a transmission line (TL), where the emitted photons propagate. We refer readers to a paper which implemented a heterodyne measurement of a KPO^28^.

In order to take into account the effect of the homodyne detection on the density matrix of the KPO $\rho _\mathrm{c}$, we use a stochastic master equation (SME) represented as^34,43^

1 $$\begin{aligned} \begin{aligned} \rho _\mathrm{{c}}(t+\tau )&= \rho _\mathrm{{c}}(t) -i \left[ - \frac{\chi }{2}\hat{a}^\dagger \hat{a}^\dagger \hat{a}\hat{a} + \beta (\hat{a}^\dagger \hat{a}^\dagger +\hat{a}\hat{a}) ,\rho _\mathrm{{c}}(t)\right] \tau +\left[ \kappa \hat{a}\rho _\mathrm{{c}}(t)\hat{a}^{\dagger } - \frac{\kappa }{2}\left\{ \hat{a}^\dagger \hat{a},\rho _\mathrm{{c}}(t)\right\} \right] \tau \\&\quad -i\sqrt{\kappa } \left[ \exp \left( -i\Theta _\mathrm{{LO}}\right) \hat{a}\rho _\mathrm{{c}}(t) - \exp \left( i\Theta _\mathrm{{LO}}\right) \rho _\mathrm{{c}}(t)\hat{a}^{\dagger } \right] \Delta W(t) -\mathrm {Tr}[\rho _\mathrm{{c}}(t)\hat{A}_{\Theta _\mathrm{{LO}}}] \rho _\mathrm{{c}}(t) \Delta W(t), \\ \end{aligned} \end{aligned}$$

where $\chi$, $\beta$ and $\kappa$ are the anharmonicity parameter of the KPO, amplitude of the pump field and the decay rate to the TL, respectively. We refer readers to, e.g., Refs. ^9,31^ for the connection between the system parameters to circuit models of KPOs. In Eq. (1), $\Theta _\mathrm{{LO}}$ is the relative phase of the classical coherent light of the local oscillator and the pump field. $\Delta W$ is the noise in the photon numbers measured by the two detectors, which is assumed to be Gaussian white noise with the mean of 0 and variance $\tau$, and $\Delta W^2=\tau$^43^. We hereafter refer to $\Delta W$ as noise. $\hat{a}$ is the annihilation operator for the KPO, and $\hat{A}_{\Theta _\mathrm{{LO}}}$ is defined by $\hat{A}_{\Theta _\mathrm{{LO}}} = i\sqrt{\kappa }[ \exp \left( i\Theta _\mathrm{{LO}}\right) \hat{a}^\dagger - \exp \left( -i\Theta _\mathrm{{LO}}\right) \hat{a}]$. The solution $\rho _\mathrm{c}(t)$ of the SME (1) for a given $\Delta W$ represents one possible realization of the dynamics under homodyne detection. The ensemble average of $\rho _\mathrm{{c}}(t)$ over $\Delta W$ in the SME (1) coincides with the density operator of the master Eq. (S2) which governs time evolution of the KPO when it is not measured (See Supplementary Section S1 for the Hamiltonian and master equation of a KPO).

Our model assumes infinitely large bandwidth of the detectors^43^, while an actual detector should have finite bandwidth. However, we consider that this will not make a considerable difference in results from measurements with finite bandwidth because we do not use an additional external field which excites the KPO and we consider a sufficiently strong pump field which narrows the spectrum of the output field^9^.

For $\kappa /4|\chi \alpha |^2 \ll 1$ , which was realized, e.g. in Ref.^9^, the stationary state of the master equation (S2) is approximately represented as $(\left| \alpha \right\rangle \left\langle \alpha \right| +\left| -\alpha \right\rangle \left\langle -\alpha \right| )/2$ with^8,44^

2 $$\begin{aligned} |\alpha |=\left( \frac{4\beta ^2-\kappa ^2/4}{\chi ^2}\right) ^{1/4} ,~~\ \mathrm{arg}[\alpha ]=\frac{1}{2}\arcsin\left( -\frac{\kappa }{4\beta }\right) . \end{aligned}$$

As shown later, the state of the KPO jumps between $\left| \alpha \right\rangle$ and $\left| -\alpha \right\rangle$. We aim at stochastically preparing either of the states using the data measured by the detectors. In order to evaluate the efficiency of the protocol, we use the fidelities defined by $F_{\pm }=\mathscr {F}[\rho _\mathrm{{c}}(t),\left| \pm \alpha \right\rangle \left\langle \pm \alpha \right| ]$, where $\mathscr {F}[\rho _\mathrm{a},\rho _\mathrm{b}]=\left( \mathrm {Tr}[\sqrt{\sqrt{\rho _\mathrm{a}}\rho _\mathrm{b} \sqrt{\rho _\mathrm{a}}}]\right) ^2$^45^. (The fidelities between the state of a KPO and these coherent states have not been examined with SME (1) to the best of our knowledge.) In numerical simulations, we assume the followings: the initial state of the KPO is $(\left| \alpha \right\rangle \left\langle \alpha \right| +\left| -\alpha \right\rangle \left\langle -\alpha \right| )/2$ to which the KPO relaxes due to the decay to the TL^44^ when the KPO is not measured; all the system parameters are fixed during the homodyne detection. We used QuTiP^46^ for a part of numerical simulations.

In Ref. ^47^, the authors aim at taking a cat state out of a KPO avoiding unwanted disturbance due to the Kerr nonlinearity. To avoid such disturbance of emitted photons, they use tailored time dependence of a pump field. On the other hand, we do not aim at taking the cat state out of a KPO, but rather aim at clarifying the correlation between measurement results and the state of the KPO from a point of view of state preparation. Therefore, we use a fixed pump field in contrast to their paper, and the Kerr nonlinearity does not cause a crucial problem to our purposes.

## Results

**Figure 2:**
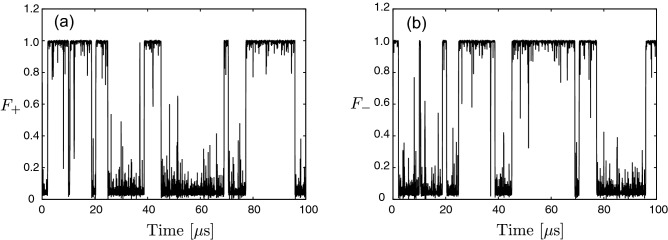
Time dependence of the fidelities, $F_{\pm }=\mathscr {F}[\rho _\mathrm{{c}}(t),\left| \pm \alpha \right\rangle \left\langle \pm \alpha \right| ]$. Panels (**a**) and (**b**) are for $F_{+}$ and $F_{-}$, respectively. The used parameters are $\chi /2\pi =3~\mathrm {MHz}$, $\beta /2\pi =3~\mathrm {MHz}$ and $\kappa /2\pi =3~\mathrm {MHz}$. $\alpha$ given by Eq. (2) is approximately $1.38-0.18i$. These parameters are experimentally feasible^9^.

Figure 2a,b shows the time dependence of the fidelities $F_\mathrm{\pm }$, where we assumed that there is no photon loss (the effect of photon loss is examined in the section entitled “Imperfect detection”). The time dependence of $F_\mathrm{\pm }$ implies that the state of the KPO jumps between $\left| \alpha \right\rangle$ and $\left| -\alpha \right\rangle$, and remains in either of the coherent states between jumps. Importantly, we cannot obtain $F_\mathrm{\pm }$ in actual measurements. In the following, we investigate how accurately we can estimate the state of the KPO from the measurement results.

### State estimation

Measurement results that observers can obtain in the homodyne detection is the difference between the numbers of photons detected by the two detectors. We use this data for the estimation of the state of the KPO. The difference between the numbers of photons detected by detectors 1 and 2 from *t* to $t+\tau$ is represented as^43,48^

3 $$\begin{aligned} \begin{aligned} \Delta N(t)=\frac{1}{\varepsilon } \left( \Delta W(t)+ \tau \mathrm {Tr}[\rho _\mathrm{{c}}\hat{A}_{\Theta _\mathrm{{LO}}}] \right) , \end{aligned} \end{aligned}$$

where $\tau$ is much smaller than $\beta ^{-1}, \chi ^{-1}$ and $\kappa ^{-1}$. Here, $\epsilon ^{-1}$ is the product of the square root of phase velocity in the TL and the intensity of the classical coherent light^43^. When the KPO is in either the two coherent states, that is, $\rho _c=\left| \pm \alpha \right\rangle \left\langle \pm \alpha \right|$, $\Delta N$ can be written as

4 $$\begin{aligned} \begin{aligned} \Delta N_\pm = \frac{1}{\epsilon } \left( \Delta W(t)\pm 2|\alpha |\sqrt{\kappa }\tau \sin (\delta \theta ) \right) \end{aligned} \end{aligned}$$

with $\delta \theta =\mathrm {arg}[\alpha ]-\Theta _{\mathrm {LO}}$. Importantly, the sign and amplitude of the second term depend on the state of the KPO and $\delta \theta$, respectively. We mainly discuss the case for $\delta \theta =\pi /2$, which maximizes the second term of Eq. (4). The effect of the deviation of $\delta \theta$ from $\pi /2$ is examined in the section entitled “Imperfect detection”.

If the amplitude of the noise $|\Delta W|$ is always smaller than $2|\alpha |\sqrt{\kappa }\tau$, we can identify the state of the KPO from the sign of $\Delta N(t)$. However, as shown below, $|\Delta W|$ can be larger than $2|\alpha |\sqrt{\kappa }\tau$. Therefore, it is important to take time average of $\Delta N(t)$ for a certain period of time to decrease the effect of the noise. The photon-number difference averaged from $t-T_\mathrm{{a}}$ to *t* is represented as

5 $$\begin{aligned} \bar{N}(t,T_\mathrm{a})=\frac{\tau }{T_\mathrm{a}} \sum _{k=0}^{T_\mathrm{a}/\tau }\Delta N (t-k\tau ), \end{aligned}$$

where we assume $T_\mathrm{a}$ is integer multiple of $\tau$.

We estimate the state of the KPO at time *t* using the sign of $\bar{N}$, that is, we estimate the KPO to be in $\left| \alpha \right\rangle \left\langle \alpha \right|$ for $\bar{N}>0$ and $\left| -\alpha \right\rangle \left\langle -\alpha \right|$ for $\bar{N}<0$, respectively. The estimated state is represented as

6 $$\begin{aligned} \rho _\mathrm{{est}}(t,T_\mathrm{a}) = \left\{ \begin{array}{ll} \left| \alpha \right\rangle \left\langle \alpha \right| &{} (\bar{N}(t,T_\mathrm{a})>0),\\ \left| -\alpha \right\rangle \left\langle -\alpha \right| &{} (\bar{N}(t,T_\mathrm{a})<0). \end{array} \right. \end{aligned}$$

**Figure 3:**
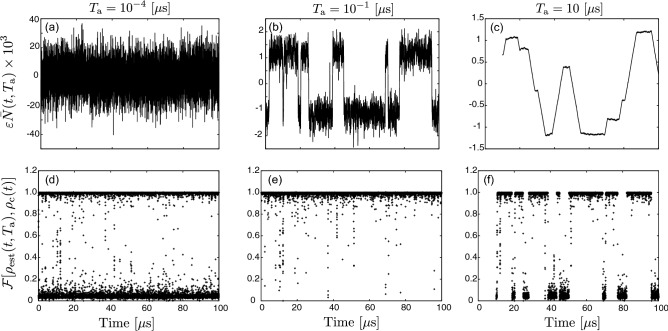
Time dependence of $\bar{N}$ and $\mathscr {F}[\rho _\mathrm{{est}}(t,T_\mathrm{a}),\rho _\mathrm{{c}}(t)]$ for $T_\mathrm{{a}}=10^{-4}~{\mu } \mathrm{s}$ (**a**,** d**), $T_\mathrm{{a}}=10^{-1}~{\mu } \mathrm{s}$ (**b**,** e**), $T_\mathrm{{a}}=10~{\mu } \mathrm{s}$ (**c**,** f**). The used parameters are the same as in Fig. 2. The data is represented by lines and dots in the upper and lower panels, respectively.

Figure 3a–c shows the time dependence of $\bar{N}$ for various values of $T_\mathrm{a}$. For $T_\mathrm{{a}}=10^{-4}$ $\mu$s, the fluctuation of $\bar{N}$ is too larger to identify the state of the KPO due to the noise (Fig. 3a). On the other hand, for $T_\mathrm{{a}}=10^{-1}$ $\mu$s, $\bar{N}$ approximately takes either of $\pm 2|\alpha |\sqrt{\kappa }\tau$ (Fig. 3b). For $T_\mathrm{{a}}=10$ $\mu$s, the second term of Eq. (4) is smeared because of bit flips and the long averaging time (Fig. 3c).

Figure 3d–f shows the fidelity of the estimation defined by $\mathscr {F}[\rho _\mathrm{{est}}(t,T_\mathrm{a}),\rho _\mathrm{{c}}(t)]$. The fidelity is close to 0 or 1 most of the time, and thus the distribution of the fidelity is bimodal. The fidelity of approximately zero corresponds to the case that the estimated state is $\left| \pm \alpha \right\rangle$ while the KPO is actually in $\left| \mp \alpha \right\rangle$. The fidelity is larger than 0.99 most of the time for $T_\mathrm{{a}}=10^{-1}$ ${\mu } \mathrm{s}$. On the other hand, the fidelity for $T_\mathrm{{a}}=10^{-4}$ $\mu$s and 10 $\mu$s can become approximately zero due to the too short and too long averaging times, respectively, thus the time-averaged fidelity is decreased.

### Proper averaging time for accurate estimation

**Figure 4:**
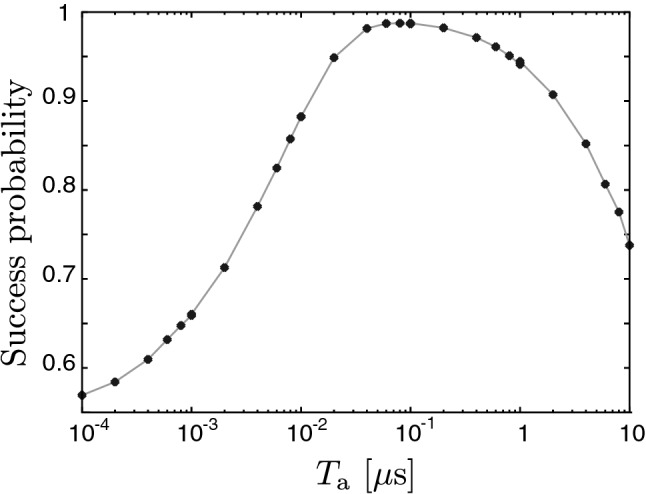
Success probability, defined by $\mathscr {F}[\rho _\mathrm{{est}}(t,T_\mathrm{a}),\rho _\mathrm{{c}}(t)]$ time-averaged over a 1000 $\mathrm \mu$s period, as a function of $T_\mathrm{a}$. The used parameters are the same as in Fig. 2.

Figure 4 shows the $T_\mathrm{a}$ dependence of $\mathscr {F}[\rho _\mathrm{{est}}(t,T_\mathrm{a}),\rho _\mathrm{{c}}(t)]$ time averaged over a $1000~\mu$s period. Hereafter, we refer to the averaged fidelity as the success probability of estimation. The success probability is higher than 0.987 around $T_\mathrm{a}=10^{-1}$ ${\mu }$s. It is clearly seen that there is a proper range of $T_\mathrm{a}$ to obtain the high success probability. The proper range of $T_\mathrm{a}$ for a given value of the success probability *K* is bounded from below due to the noise and bounded from above due to smearing by the time averaging. In the following, we obtain the upper bound $T_{{K}}^\mathrm{{U}}$ and lower bound $T_{{K}}^\mathrm{{L}}$ of $T_\mathrm{{a}}$ for a given success probability *K*.

#### Lower bound $T_{K}^\mathrm{{L}}$

**Figure 5:**
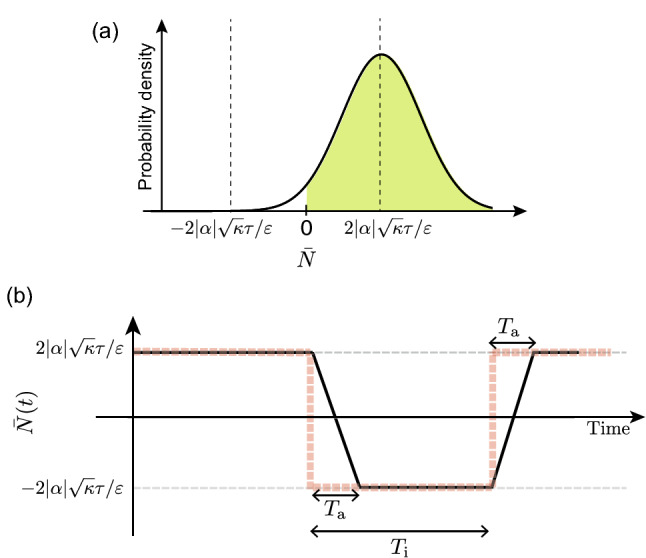
Panel (**a**): Schematic illustration of the distribution of $\bar{N}$ fluctuating around $\Delta N'=2|\alpha |\sqrt{\kappa }\tau /\varepsilon$. The ratio of the colored area to the total area under the Gaussian curve is the same as the success probability *K*. Panel (**b**): Schematic illustration of a typical time evolution of $\bar{N}$ in the binomial-coherent-state model in which the noise is neglected. The dashed and solid lines represent $\bar{N}$ for $T_\mathrm{{a}}=0$ (without time averaging) and $0<T_\mathrm{{a}}<T_\mathrm{{i}}$ (with time averaging), respectively.

We consider the case that the averaging time $T_\mathrm{a}$ is much shorter than the typical duration in which the KPO remains in either of $\left| \pm \alpha \right\rangle$. Then, $\Delta {N}(t)$ is typically represented as Eq. (4) and fluctuates around either of $\pm 2|\alpha |\sqrt{\kappa }\tau /\varepsilon$ due to the Gaussian noise $\Delta W$, except when jumps occur. The fluctuation of $\bar{N}(t)$ has the Gaussian distribution with the standard deviation of $\sigma (T_\mathrm{a}) = \sqrt{{\tau ^2}/{T_\mathrm{a}\varepsilon ^2}}$, where the effect of jumps to $\bar{N}(t)$ is neglected because jumps seldom occur in $T_\mathrm{a}$. Then, the success probability *K* can be related to $T_\mathrm{a}$ as

7 $$\begin{aligned} K = \int _{-2|\alpha |\sqrt{\kappa }\tau /\varepsilon }^{\infty } dx \frac{1}{\sigma (T_\mathrm{a})\sqrt{2\pi }} \exp \Big [\frac{-x^2}{2\sigma ^2(T_\mathrm{{a}})}\Big ], \end{aligned}$$

where *K* is the same as the ratio of the colored area to the total area under the Gaussian curve illustrated in Fig. 5a. $T_\mathrm{a}$ in Eq (7) equals to the lower bound of the averaging time, $T_{K}^\mathrm{{L}}$, to obtain the success probability higher than or equal to *K*. Therefore, we can obtain $T_{K}^\mathrm{{L}}$ by solving Eq. (7). For example, $T_{K}^\mathrm{{L}}$ for $K=0.95$ is

8 $$\begin{aligned} \begin{aligned} T_{0.95}^\mathrm{{L}}=\frac{1.65^2}{4|\alpha |^2 \kappa } . \end{aligned} \end{aligned}$$

#### Upper bound $T_{K}^\mathrm{{U}}$

Averaging over a long period of time can degrade the accuracy of the estimation of the state of the KPO due to the jump. We assume that this smearing effect determines $T_{K}^\mathrm{{U}}$, and derive $T_{K}^\mathrm{{U}}$ by developing a binomial-coherent-state model that describes the stochastic dynamics of the KPO in Eq. (1).

In the binomial-coherent-state model, we assume that the state of the KPO can only take either of $\left| \pm \alpha \right\rangle$, and jumps between them with a probability of $p~(=\Omega dt)$ in a small time *dt*. This stochastic process can be represented as the binomial process in the two coherent states. Then, the mean time interval between jumps is

9 $$\begin{aligned} E[T_\mathrm{{i}}]=\frac{1}{\Omega }, \end{aligned}$$

because $\Omega$ is the average rate of jumps. Figure 5b illustrates a typical time evolution of $\bar{N}$. Due to smearing effect and jumps, wrong estimations occur in the period of $T_\mathrm{{a}}/2$ per jump. The error rate, defined by the ratio of the duration of the wrong estimation to the total measurement time, can be written as $T_\mathrm{{a}}/2E[T_\mathrm{{i}}]$, and the error rate is also written as $1-K$. Thus, we obtain $1-K=T_{K}^\mathrm{{U}}/2E[T_\mathrm{{i}}]$, where we replaced $T_\mathrm{{a}}$ by $T_{K}^\mathrm{{U}}$. Using Eq. (9), we obtain $T_{K}^\mathrm{{U}}$ as

10 $$\begin{aligned} T_{K}^\mathrm{{U}} =2(1-K)/\Omega . \end{aligned}$$

Now, we obtain $\Omega$ by the following manner. In the binomial-coherent-state model, the ensemble average of the density operator can be represented as

11 $$\begin{aligned} \bar{\rho }_\mathrm{b}(t)= \sum _{k=2n}^{N}{}_N \mathrm {C}_k p^{k} (1-p)^{N-k} \left| \alpha \right\rangle \left\langle \alpha \right| + \sum _{k=2n+1}^{N}{}_N \mathrm {C}_k p^{k} (1-p)^{N-k} \left| -\alpha \right\rangle \left\langle -\alpha \right| , \end{aligned}$$

where $N=t/dt$, and we assumed that the KPO is in $\left| \alpha \right\rangle$ at the initial time. As shown in Supplementary Section S2, the expectation value of $\hat{x}=(\hat{a}+\hat{a}^\dagger )/2$ corresponding to $\bar{\rho }_\mathrm{b}(t)$ in Eq. (11) is written as $\langle \hat{x} \rangle = \mathrm{Re}[\alpha ] \exp (-2\Omega t)$ in the limit of $dt\rightarrow 0$. Because the binomial-coherent-state model approximates the dynamics governed by the SME, $\bar{\rho }_\mathrm{b}(t)$ approximately coincides with the solution of the master equation (S2) (Note that the ensemble average of $\rho _\mathrm{{c}}(t)$ over $\Delta W$ coincides with the density operator of the master equation). Therefore, we can obtain $\Omega$ by fitting $\mathrm{Re}[\alpha ] \exp (-2\Omega t)$ to ${\langle \hat{x}\rangle }$ with the master equation (S2) (the detailed discussion and results of the fitting can be found in Supplementary Section S2).

In Ref. ^9^, it is shown that the effect of the quantum jumps between $\left| \alpha \right\rangle$ and $\left| -\alpha \right\rangle$ manifests itself as finite width of a peak in power spectral density because the width is determined by the timescale of the fluctuations in the phase of the state of a KPO. The phase fluctuation is suppressed with the increase of the pump amplitude^49^. On the other hand, in our simulation the jumps are observed more directly in the difference between the numbers of photons detected by detectors 1 and 2, $\Delta N(t)$. The jump rate determines the upper bound of averaging time to obtain high success probability.

#### Numerical results

**Figure 6:**
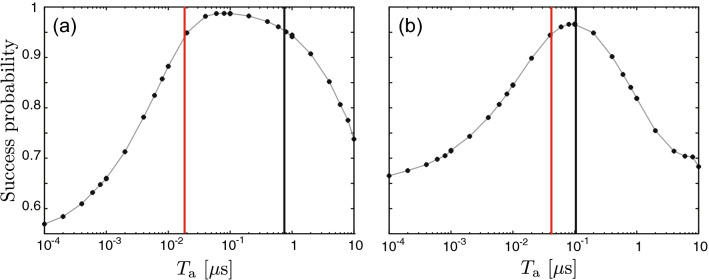
$T_\mathrm{a}$ dependence of the mean of $\mathscr {F}[\rho _\mathrm{{est}}(t,T_\mathrm{a}),\rho _\mathrm{{c}}(t)]$ for $\kappa =\chi$, $\beta =\chi$ (**a**) and $\kappa =\chi$, $\beta =\chi /2$ (**b**). The parameters for panels (**a**) and (**b**) correspond $\alpha =1.38-0.18i$ and $\alpha =0.90-0.24i$, respectively. The vertical red (black) line represents $T_{0.95}^\mathrm{{L(U)}} = 1.86\times 10^{-2}~{\mu } \mathrm{s}~(7.52\times 10^{-1}~{\mu } \mathrm{s})$ in panel (**a**) and $T_{0.95}^\mathrm{{L(U)}} =4.17\times 10^{-2}~{\mu } \mathrm{s}~(1.04\times 10^{-1}~{\mu } \mathrm{s})$ in panel (**b**). The other parameters are the same as in Fig. 2.

Figure 6 shows the $T_\mathrm{a}$ dependence of the success probability together with $T_{0.95}^\mathrm{{L(U)}}$ for two different parameter sets. It is seen that the values of $T_\mathrm{{K}}^\mathrm{{L(U)}}$ obtained in the above section approximate well the numerical ones.

**Figure 7:**
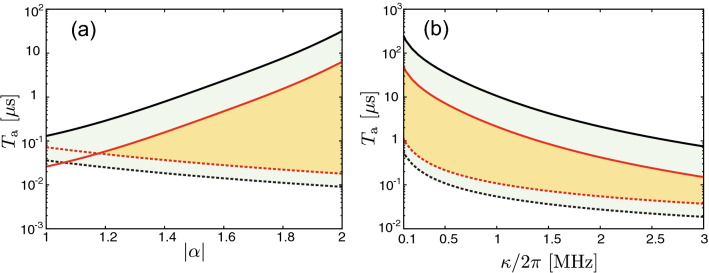
(**a**) $T_{K}^\mathrm{U}$ and $T_{K}^\mathrm{L}$ as a function of $|\alpha |$ in Eq. (2). The solid and dashed lines are for $T_{K}^\mathrm{U}$ and $T_{K}^\mathrm{L}$, respectively. The black and red data are for $K=0.95$ and 0.99, respectively, where $\beta$ was changed, while *K* is fixed, in order to change $|\alpha |$. (**b**) The same things as (**a**) but as a function of $\kappa$. The other parameters are the same as in Fig. 2.

Figure 7a represents the high success probability regime in the $T_\mathrm{a}$-$|\alpha |$ plane. It is seen that $T_K^\mathrm{L}$ decreases with the increase of $|\alpha |$ as analytically exemplified in Eq. (8) because the effect of the noise to the result of the estimation becomes small for large $|\alpha |$. On the other hand, $T_K^\mathrm{U}$ increases with $|\alpha |$ because $E[T_\mathrm{i}]$ increases exponentially with $|\alpha |^2$ ^50^ as shown in Supplementary Section S2. Thus, the range of $T_\mathrm{a}$, which gives the high success probability, increases with $|\alpha |$. The maximum success probability also increases with $|\alpha |$. We attribute this to the fact that the two quasi stable states, between which the KPO jumps, can be approximated by $\left| \pm \alpha \right\rangle$ more accurately in Eq. (2) when $|\alpha |$ increases^21^.

We examine the proper range of the averaging time for smaller photon decay rate $\kappa$. Figure 7b represents the high success probability regime in the $T_\mathrm{a}$-$\kappa$ plane. It is seen that $T_K^\mathrm{L}$ increases with the decrease of $\kappa$ because the output field from the KPO becomes weak as $\kappa$ decreases. This implies that we need longer averaging time due to the weaker output field for smaller $\kappa$. However small $\kappa$ rather enlarges the proper range of the averaging time because $T_{K}^\mathrm{U}$ increases with the decrease of $\kappa$ due to lengthened $E[T_i]$ (see Supplementary Section S2).

### Imperfect detection

In the previous sections, we considered the ideal homodyne detection without photon loss, and we set $\delta \theta =\pi /2$ in order to maximize the amplitude of the second term of Eq. (4). In this section, we examine the effect of the photon loss and the deviation of $\delta \theta$ from the ideal value on the success probability of estimation.

We consider the case that a proportion $\eta$ of photons are detected, while the rest are lost. We assume that photons leaked from the KPO do not return to the KPO. We refer $\eta$ as the efficiency of the measurement. For the measurement with the efficiency $\eta$, the SME and measurement result are represented as^43,51^

12 $$\begin{aligned} \begin{aligned} \rho _\mathrm{{c}}(t+\tau )&= \rho _\mathrm{{c}}(t) -i \left[ \left( \omega _{s}-\chi -\frac{\omega _p}{2}\right) \hat{a}^\dagger \hat{a} - \frac{\chi }{2}\hat{a}^\dagger \hat{a}^\dagger \hat{a}\hat{a} + \beta (\hat{a}^\dagger \hat{a}^\dagger +\hat{a}\hat{a}) ,\rho _\mathrm{{c}}(t)\right] \tau \\&\quad +\left[ \kappa _\mathrm{ex}\hat{a}\rho _\mathrm{{c}}(t)\hat{a}^{\dagger } - \frac{\kappa _\mathrm{ex}}{2}\left\{ \hat{a}^\dagger \hat{a},\rho _\mathrm{{c}}(t)\right\} \right] \tau \\&\quad -i\sqrt{\kappa _\mathrm{ex}} \left[ \exp \left( -i\Theta _\mathrm{LO}\right) \hat{a}\rho _\mathrm{{c}}(t) - \exp \left( i\Theta _\mathrm{LO}\right) \rho _\mathrm{{c}}(t)\hat{a}^{\dagger } \right] \sqrt{\eta }\Delta W(t) \\&\quad -\mathrm {Tr}[\rho _\mathrm{{c}}(t)\hat{A}_{\Theta _\mathrm{LO}}] \rho _\mathrm{{c}}(t) \sqrt{\eta }\Delta W(t), \end{aligned} \end{aligned}$$

and

13 $$\begin{aligned} \begin{aligned} \Delta N(t)=\frac{1}{\varepsilon } \left( \sqrt{\eta }\Delta W+ \eta \tau \mathrm {Tr}[\rho _\mathrm{{c}}\hat{A}_{\Theta _\mathrm{LO}}] \right) . \end{aligned} \end{aligned}$$

As in the previous section, we assume that the averaging time $T_\mathrm{a}$ is much shorter than the typical duration that the KPO remains in either of $\left| \pm \alpha \right\rangle$. When $\rho _\mathrm{{c}}=\left| \pm \alpha \right\rangle \left\langle \pm \alpha \right|$, $\Delta N$ fluctuates around $\pm 2|\alpha |\sqrt{\kappa }\tau \sin (\delta \theta )\eta /\varepsilon$; the standard deviation of $\bar{N}$ is $\sigma (T_\mathrm{{a}},\eta )=\sqrt{{\tau ^2\eta }/{T_\mathrm{{a}}\varepsilon ^2}}$. The success probability *K* can be related to $T_\mathrm{a}$ as

14 $$\begin{aligned} K = \int _{-2|\alpha |\sqrt{\kappa }\tau \sin (\delta \theta ) \eta /\varepsilon }^{\infty } dx \frac{1}{\sigma (T_\mathrm{a},\eta )\sqrt{2\pi }} \exp \Big [\frac{-x^2}{2\sigma ^2(T_\mathrm{{a}},\eta )}\Big ]. \end{aligned}$$

We can obtain $T_{K}^\mathrm{{L}}$ by solving Eq. (14). For example, $T_{K}^\mathrm{{L}}$ for $K=0.95$ is

15 $$\begin{aligned} \begin{aligned} T_{0.95}^\mathrm{{L}}=\frac{1.65^2}{4|\alpha |^2 \kappa \sin ^2(\delta \theta )\eta } . \end{aligned} \end{aligned}$$

On the other hand, $T_{K}^\mathrm{{U}}$ in Eq. (10) does not depend on $\eta$ and $\delta \theta$ because it is derived by using the master equation (S2) that does not have $\eta$ and $\delta \theta$.

**Figure 8:**
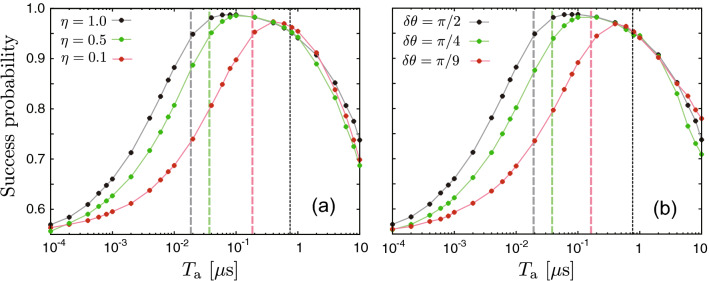
Dependence of the success probability on $T_\mathrm{a}$ for various values of $\eta$ and $\delta \theta$. Panel (**a**) is for $\delta \theta =\pi /2$; panel (**b**) is for $\eta =1$. The other parameters are the same as in Fig. 2. In panel (**a**), the vertical gray, green and red dashed lines represent $T_{0.95}^\mathrm{L}=1.86\times 10^{-2}$, $3.73\times 10^{-2}$ and $1.86\times 10^{-1}~{\mu } \mathrm{s}$, respectively; in panel (**b**), the vertical gray, green and red dashed lines represent $T_{0.95}^\mathrm{L}=1.86\times 10^{-2}$, $3.73\times 10^{-2}$ and $1.59\times 10^{-1}~{\mu } \mathrm{s}$, respectively. The vertical black dotted line is for $T_{0.95}^\mathrm{U}=7.52\times 10^{-1}~{\mu } \mathrm{s}$ in both panels.

Figure 8 shows the dependence of the success probability on $T_\mathrm{a}$ for various values of $\eta$ and $\delta \theta$ with $T_{K}^\mathrm{{L}}$ and $T_{K}^\mathrm{{U}}$. The success probability decreases on the left side of its peak as $\eta$ decreases or $\delta \theta$ deviates from $\pi /2$. On the other hand, the right side of the peak is not sensitive to $\eta$ and $\delta \theta$. These results are consistent with the analysis of $T_{K}^\mathrm{{L}}$ and $T_{K}^\mathrm{{U}}$.

## Conclusions and discussions

We have studied the stochastic state preparation of a KPO based on homodyne detection. We have shown that the measured data, if it is time averaged with a proper averaging time to decrease the effect of noise, has a strong correlation with the state of the KPO, and therefore can be used for estimation of the state of the KPO. We have quantitatively examined the success probability of the estimation taking into account the effect of the noise and bit flips, and have shown that the success probability is higher than 0.987 with the parameter used. We have developed a binomial-coherent-state model, which describes the stochastic dynamics of the KPO under homodyne detection, and by using it we have obtained a proper range of the averaging time to realize the high success probability. Our analysis based on the binomial-coherent-state model implies that the success probability is further increased as the size of the coherent state becomes large. Furthermore, we have examined the effect of the imperfection of the measurement and the choice of the phase of the coherent light of the local oscillator, on the state estimation. Our scheme of state preparation of KPOs does not require a drive field nor modulation of the pump field in contrast to conventional methods.

In Supplementary Section S3, we examine alternative protocols, using temporal controls of system parameters, for preparation of pure states of a KPO. The protocol with an auxiliary time dependent drive field can give slightly higher success probability than our method when the phase and time dependence of the amplitude of the drive field are properly chosen. However, finding the proper parameters will actually require experimental efforts. Despite its simplicity, the measurement-based protocol can give success probability comparable to that of the protocol with a temporally controlled drive field. Moreover, our method can give higher success probability than the method to create a cat state for the used parameters.

Although we focused on preparation of the two stable coherent states in this paper, this method followed by single-qubit gates conditioned on measurement results can generate an arbitrary qubit state. Our scheme of state preparation can be applied straightforwardly to multi-KPO systems when the time interval of jumps of KPOs is sufficiently long. It is also expected that turning on the ferromagnetic or anti-ferromagnetic coupling between KPOs^42^ can increase the efficiency of state preparation by mitigating bit flips of individual KPOs.

## Supplementary Information

Supplementary Information.

## Data Availability

All data generated or analysed during this study are included in this published article and its Supplementary Information files.
