# Supplementary material for: Measurement-based preparation of stable coherent states of a Kerr parametric oscillator
Source: Sci Rep. 2023 Jan 28;13:1606. doi: 10.1038/s41598-023-28682-1 (PMC9884232; doi:10.1038/s41598-023-28682-1)
Supplement: Supplementary file 1 — Supplementary Information. [file 41598_2023_28682_MOESM1_ESM.pdf]

Supplemental information: Measurement-based preparation of stable coherent states of a Kerr parametric oscillator

Yuta Suzuki[1,2], Shohei Watabe[1,3], Shiro Kawabata[2,4] and Shumpei Masuda[2,4,*]

[1] Department of Physics, Faculty of Science Division I, Tokyo University of Science, 1-3 Kagurazaka, Shinjuku-ku, Tokyo 162-8601, Japan.
[2] Research Center for Emerging Computing Technologies (RCECT), National Institute of Advanced Industrial Science and Technology (AIST), 1-1-1, Umezono, Tsukuba, Ibaraki 305-8568, Japan.
[3] College of Engineering, Department of Computer Science and Engineering, Shibaura Institute of Technology, 3-7-5 Toyosu, Koto-ku, Tokyo 135-8548, Japan
[4] NEC-AIST Quantum Technology Cooperative Research Laboratory, National Institute of Advanced Industrial Science and Technology (AIST), Tsukuba, Ibaraki 305-8568, Japan.

* shumpei.masuda@aist.go.jp

S1 Hamiltonian and master equation

The Hamiltonian for the composite system of a Kerr parametric oscillator (KPO) and a transmission line (TL) can be written as

$$\hat{H}_{\rm tot} = \hbar\omega_s\hat{a}^\dagger\hat{a} - \frac{\hbar\chi}{12}(\hat{a}^\dagger+\hat{a})^4 + 2\hbar\beta(\hat{a}^\dagger+\hat{a})^2\cos(\omega_p t) + \hbar\int_0^\infty dk v_b k \hat{b}_k^\dagger\hat{b}_k + \hbar\sqrt{\frac{v_b\kappa}{2\pi}}\int_0^\infty dk\left(\hat{a}^\dagger\hat{b}_k + \hat{b}_k^\dagger\hat{a}\right), \tag{S1}$$

where ω_s is the resonance frequency of the KPO when no pump filed is applied, and $\hat{a}$ is the annihilation operator for the KPO. The second and third terms represent the anharmonicity of the KPO and the effect of the pump[1,2], respectively. β, ω_p and χ are the amplitude and angular frequency of the pump and the anharmonicity parameter of the KPO, respectively. The fourth term is the Hamiltonian of the eigenmodes of the TL, and the fifth term is the interaction Hamiltonian between the KPO and TL. Here, $\hat{b}_k$ is the annihilation operator of the mode with wave number k in the TL; v_b is the phase velocity of the microwave in the TL; κ is the decay rate to the TL. We assume that the loss of microwave photons is negligible for simplicity.

In a frame rotating at $\omega_p/2$, the master equation for the KPO is represented as[1]

$$\frac{d\rho(t)}{dt} = -i\left[\Delta\hat{a}^\dagger\hat{a} - \frac{\chi}{2}\hat{a}^\dagger\hat{a}^\dagger\hat{a}\hat{a} + \beta(\hat{a}^\dagger\hat{a}^\dagger+\hat{a}\hat{a}),\rho(t)\right] + \left[\kappa_{\rm ex}\hat{a}\rho(t)\hat{a}^\dagger - \frac{\kappa_{\rm ex}}{2}\left\{\hat{a}^\dagger\hat{a},\rho(t)\right\}\right], \tag{S2}$$

where ρ is the density operator, and $\Delta = \omega_s - \chi - \omega_p/2$. The steady state of the master equation (S2) is approximated by the completely mixed state $(|\alpha\rangle\langle\alpha| + |-\alpha\rangle\langle-\alpha|)/2$ of the coherent states $|\alpha\rangle$ and $|-\alpha\rangle$, where α is given by Eq. (2).

S2 Average of jump interval

We obtain the average of the time interval between jumps, $E[T_{\rm i}]$, by using the binomial-coherent-state model. As explained in the main text, in this model, the KPO can only take either of $|\pm\alpha\rangle$ and jumps between the two states with a probability of $p = \Omega dt$ in time dt, where Ω is the rate of jumps. The average of the time interval between jumps is given by Eq. (9). Using

Eq. (11), the expected value of $\hat{x} = (\hat{a} + \hat{a}^\dagger)/2$ is represented as

$$
\begin{aligned}
\langle \hat{x} \rangle &= \sum_{k=2n}^{N} {}_N\mathrm{C}_k p^k (1-p)^{N-k} \mathrm{Tr}[\rho(t) \left|\alpha\right\rangle \left\langle\alpha\right|] + \sum_{k=2n+1}^{N} {}_N\mathrm{C}_k p^k (1-p)^{N-k} \mathrm{Tr}[\rho(t) \left|-\alpha\right\rangle \left\langle-\alpha\right|] && \text{(S3)} \\
&= \sum_{k=2n} {}_N\mathrm{C}_k p^k (1-p)^{N-k} \mathrm{Re}[\alpha] + \sum_{k=2n+1} {}_N\mathrm{C}_k p^k (1-p)^{N-k} \mathrm{Re}[-\alpha] && \text{(S4)} \\
&= \sum_{k=2n} {}_N\mathrm{C}_k p^k (1-p)^{N-k} \mathrm{Re}[\alpha] - \sum_{k=2n+1} {}_N\mathrm{C}_k p^k (1-p)^{N-k} \mathrm{Re}[\alpha] && \text{(S5)} \\
&= \sum_{k=2n} {}_N\mathrm{C}_k (-1)^k p^k (1-p)^{N-k} \mathrm{Re}[\alpha] + \sum_{k=2n+1} {}_N\mathrm{C}_k (-1)^k p^k (1-p)^{N-k} \mathrm{Re}[\alpha] && \text{(S6)} \\
&= \mathrm{Re}[\alpha] \sum_k {}_N\mathrm{C}_k (-p)^k (1-p)^{N-k} && \text{(S7)} \\
&= \mathrm{Re}[\alpha] (-p+1-p)^N && \text{(S8)} \\
&= \mathrm{Re}[\alpha] (1-2p)^N, && \text{(S9)}
\end{aligned}
$$

where we used $N = t/dt$. Taking the limit of $dt \to 0$, we obtain

$$
\begin{aligned}
\lim_{dt \to 0} \langle \hat{x} \rangle &= \lim_{dt \to 0} \mathrm{Re}[\alpha](1-2p)^N \\
&= \lim_{dt \to 0} \mathrm{Re}[\alpha](1-2\Omega dt)^{t/dt} \\
&= \mathrm{Re}[\alpha] \exp(-2\Omega t).
\end{aligned} \tag{S10}
$$

We can obtain Ω by fitting $\langle \hat{x} \rangle$ in Eq. (S10) to the counterpart of the dynamics governed by the master equation (S2). (Note that $\rho(t)$ in Eq. (11) coincides with the solution of the master equation in Eq. (S2) when the binomial-coherent-state model is valid as explained in the main text.) Figure S1(a) presents the result of the fitting for $\alpha = 1.38 - 0.18i$ as an example. The time dependence of $\langle \hat{x} \rangle$ in Eq. (S10) with $\Omega/2\pi = 20$kHz matches well to the one obtained by solving the master equation (S2) for $\alpha = 1.38 - 0.18i$. Figure S1(b) shows $E[T_\mathrm{i}]$ as a function of $|\alpha|^2$. It is seen that $E[T_\mathrm{i}]$ exponentially increases with the increase of $|\alpha|^2$ [3]. Figure S1(c) shows $E[T_\mathrm{i}]$ as a function of κ. It is seen that $E[T_\mathrm{i}]$ rapidly increases with the decrease of κ.

S3 Other protocols

We examine alternative methods to generate pure states of a KPO, a stable coherent state and a cat state.

Stable coherent state

Stable coherent states can be generated with a controlled single-photon drive field and a fixed pump field. The role of the drive field is to tilt the effective potential of a KPO[4] in order to increase the probability of realization of $|\alpha\rangle$[5]. The time dependence of the Rabi frequency of the drive field is given by

$$
\Omega(t) = \begin{cases}
\frac{\Omega_0}{2}\left[1 - \cos\left(\frac{\pi t}{T_\mathrm{dr}^{(1)}}\right)\right] & (0 \le t \le T_\mathrm{dr}^{(1)}), \\
\Omega_0 & (T_\mathrm{dr}^{(1)} < t \le T_\mathrm{dr}^{(2)}), \\
\frac{\Omega_0}{2}\left[1 + \cos\left(\frac{\pi (t - T_\mathrm{dr}^{(2)})}{\Delta T_\mathrm{dr}}\right)\right] & (T_\mathrm{dr}^{(2)} < t \le T_\mathrm{dr}^{(3)}), \\
0 & (t > T_\mathrm{dr}^{(3)})
\end{cases} \tag{S11}
$$

where $\Delta T_\mathrm{dr} \equiv T_\mathrm{dr}^{(3)} - T_\mathrm{dr}^{(2)}$. The Rabi frequency of the drive field is gradually ramped for $0 \le t \le T_\mathrm{dr}^{(1)}$ and fixed for $T_\mathrm{dr}^{(1)} < t \le T_\mathrm{dr}^{(2)}$, and then is decreased to zero for $T_\mathrm{dr}^{(2)} < t \le T_\mathrm{dr}^{(3)}$. We set $T_\mathrm{dr}^{(1)}$ and $T_\mathrm{dr}^{(2)}$ to be long enough so that a stable coherent state is realized as the stationary state at $t = T_\mathrm{dr}^{(2)}$. We define the success probability of the preparation by the fidelity, $\mathscr{F}[\left|\alpha\right\rangle \left\langle\alpha\right|, \rho(T_\mathrm{dr}^{(3)})]$, corresponding to the time when the drive field is off because normally a KPO is not subject to a drive field unless a single-qubit gate is operated. We simulate the dynamics of the KPO using the master equation represented as[1]

$$
\frac{d\rho(t)}{dt} = -i\left[\Delta \hat{a}^\dagger \hat{a} - \frac{\chi}{2}\hat{a}^\dagger\hat{a}^\dagger\hat{a}\hat{a} + \beta(\hat{a}^\dagger\hat{a}^\dagger + \hat{a}\hat{a}) + \Omega(t)(\hat{a}^\dagger e^{-i\theta_\mathrm{dr}} + \hat{a} e^{i\theta_\mathrm{dr}}), \rho(t)\right] + \left[\kappa_\mathrm{ex}\hat{a}\rho(t)\hat{a}^\dagger - \frac{\kappa_\mathrm{ex}}{2}\left\{\hat{a}^\dagger\hat{a}, \rho(t)\right\}\right], \tag{S12}
$$

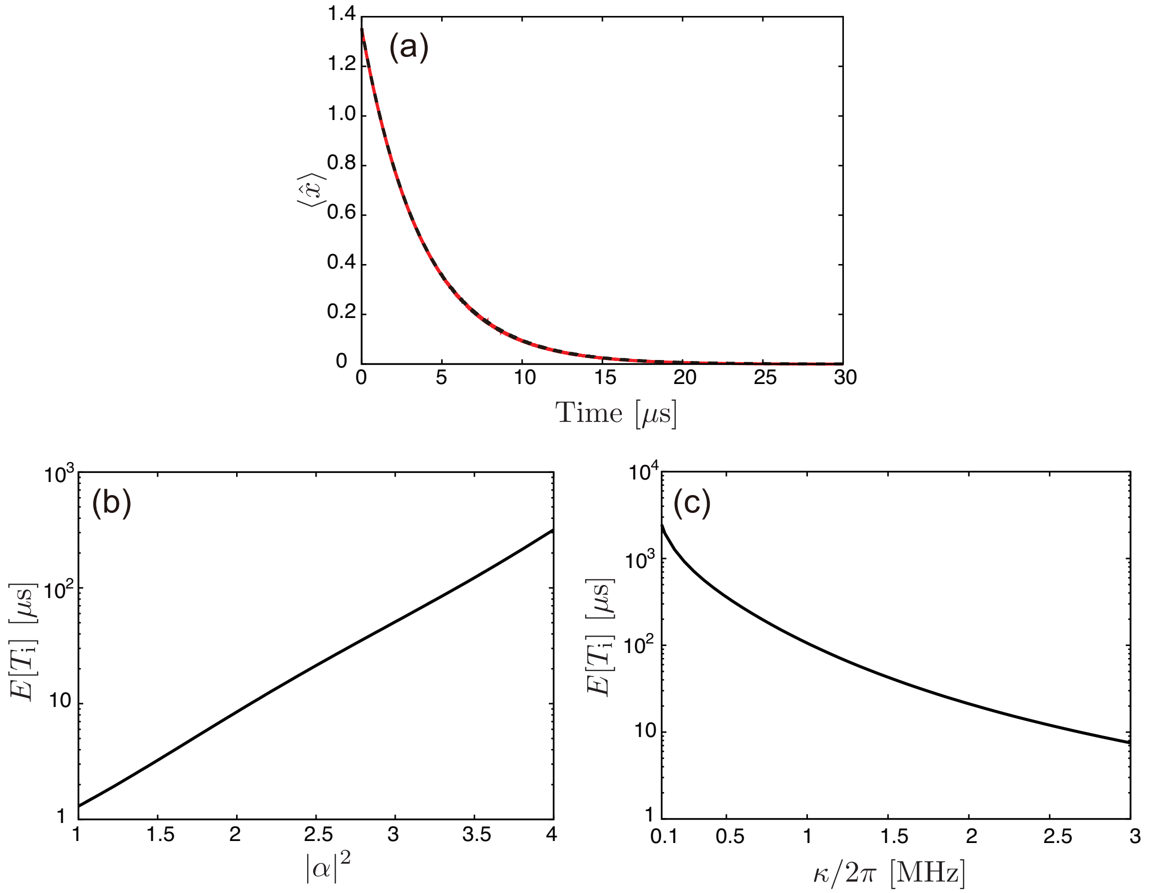

Figure S1. (a): $\langle\hat{x}\rangle$ obtained by solving the master equation (S2) for $\alpha = 1.38 - 0.18i$ (black dashed curve) and $\langle\hat{x}\rangle$ in Eq. (S10) for $\Omega/2\pi = 20$ kHz (red solid curve). The two curves are almost overlapping. (b): $E[T_{\rm i}]$ in Eq. (9) with Ω obtained by the fitting of $\langle\hat{x}\rangle$ in Eq. (S10) to numerical results of the master equation (S2). ($E[T_{\rm i}]$ is shown in a log scale.) β was changed to vary $|\alpha|$, while χ is fixed. (c): $E[T_{\rm i}]$ as a function of κ. The other parameters are the same as in Fig. 2.

.

where $\theta_{\rm dr}$ is the relative phase of the drive field to the one of the pump field[5]. We call $\theta_{\rm dr}$ the phase of the drive field for simplicity of notation. The initial state is the stationary state under the fixed pump field approximated by $(|\alpha\rangle\langle\alpha| + |-\alpha\rangle\langle-\alpha|)/2$.

Typical time dependence of Ω and the corresponding fidelity are shown in Fig. S2(a). The fidelity tends to decrease for $t > T_{\rm dr}^{(2)}$ due to bit-flip, while there is a peak just after $t = T_{\rm dr}^{(2)}$. We attribute this rise of the fidelity to that the coherent state pushed by the drive field becomes close to the target one temporally while Ω is decreased. The dependence of the success probability on $\Delta T_{\rm dr}$ is presented in Fig. S2(b,c) for various values of Ω_0. It is seen that there is a proper range of $\Delta T_{\rm dr}$ to obtain high success probability. The maximum success probability is approximately 0.993 and is slightly higher than that of our simple measurement-based protocol presented in the main text. We attribute the decrease of the success probability in the small and large-$\Delta T_{\rm dr}$ regimes to nonadiabatic transitions and bit-flip, respectively. Because the orientation of the tilting of the effective potential changes with $\theta_{\rm dr}$, the success probability depends on $\theta_{\rm dr}$. Figure S2(d) shows $\Delta T_{\rm dr}$ dependence of $1-$*success probability* for various values of $\theta_{\rm dr}$ with $\Omega_0/2\pi = 6$ MHz. It is seen that the success probability can decrease when $\theta_{\rm dr}$ deviates from zero. Therefore, both $\Delta T_{\rm dr}$ and $\theta_{\rm dr}$ should be properly chosen for high success probability.

Although we study here the protocol which does not use control of the pump field, the stable coherent state can also be generated by controlling both the drive and pump fields[5].

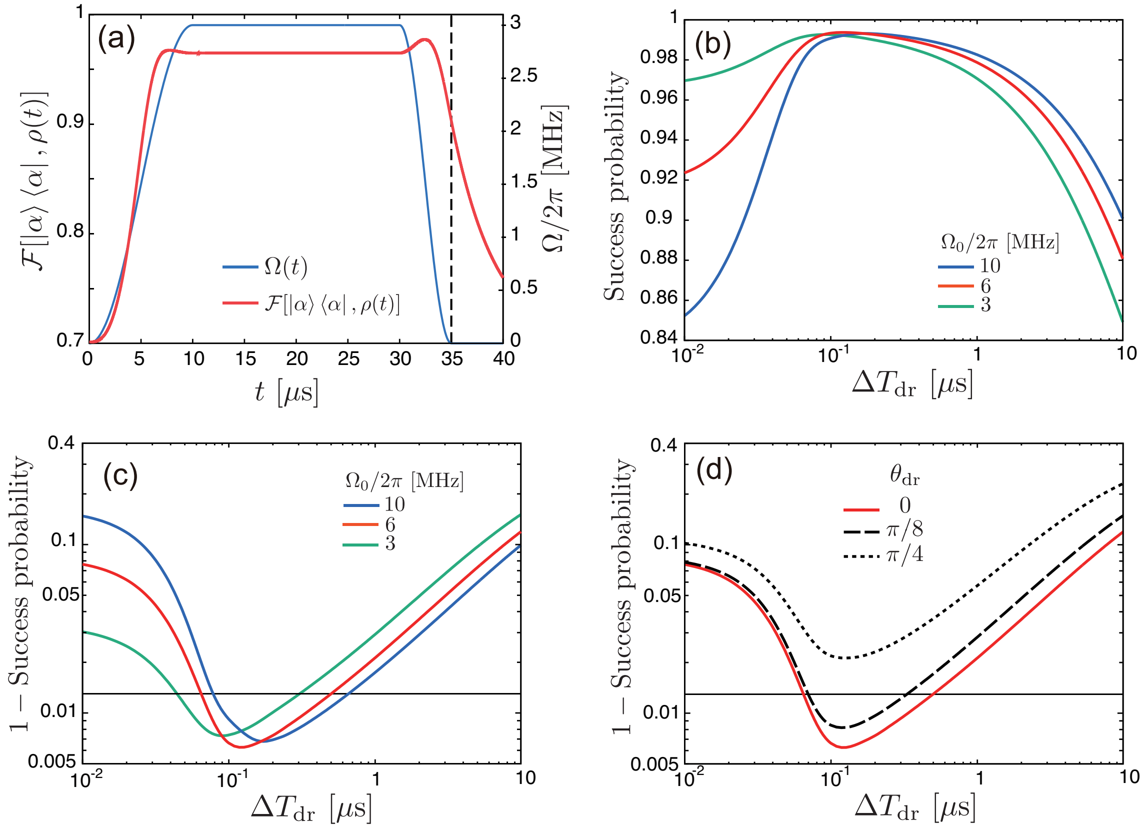

Figure S2. (a): Time dependence of Ω and the corresponding fidelity $\mathscr{F}[|\alpha\rangle\langle\alpha|, \rho(t)]$ for $T_{\rm dr}^{(1)} = 10\ \mu$s, $T_{\rm dr}^{(2)} = 30\ \mu$s, $T_{\rm dr}^{(3)} = 35\ \mu$s, $\theta_{\rm dr} = 0$ and $\Omega_0/2\pi = 3$ MHz. The dashed line indicates $t = T_{\rm dr}^{(3)}$. (b): $\Delta T_{\rm dr}$ dependence of the success probability defined by fidelity, $\mathscr{F}[|\alpha\rangle\langle\alpha|, \rho(T_{\rm dr}^{(3)})]$ for various values of Ω_0. The used $T_{\rm dr}^{(1,2)}$ and $\theta_{\rm dr}$ are the same as (a). (c): The same results as panel (b) but $1-$*success probability* in logarithmic scale. (d): $\Delta T_{\rm dr}$ dependence of $1-$*success probability* in logarithmic scale for various values of $\theta_{\rm dr}$ with $\Omega_0/2\pi = 6$ MHz. The used $T_{\rm dr}^{(1,2)}$ are the same as (a). The horizontal line in panels (c) and (d) indicates the success probability of the method based on homodyne detection. The other parameters used are the same as in Fig. 2.

.

Cat state

A cat state, which is a superposition of the stable coherent states, can be generated by gradually ramping the pump field against the vacuum state[1]. The cat state is represented as $|\Psi_{\rm cat}\rangle = N_{\rm cat}(|\alpha\rangle + |-\alpha\rangle)$ with the normalization factor $N_{\rm cat}$. The ramp

of the pump field should be slow enough because the protocol has recourse to quantum adiabatic dynamics. We numerically examine the efficiency of the control. The time dependence of the pump amplitude β is given by

$$\beta(t) = \begin{cases} \frac{\beta_0}{2}\left[1-\cos\left(\frac{\pi t}{T_{\rm p}}\right)\right] & (0 \le t \le T_{\rm p}), \\ \beta_0 & (t > T_{\rm p}), \end{cases} \tag{S13}$$

where T_p is the duration of the ramp of the pump field. The dynamics is simulated using the master equation (S2). The initial state is the vacuum state. A typical time dependence of the amplitude of the pump field and the corresponding fidelity $\mathscr{F}[|\Psi_{\rm cat}\rangle\langle\Psi_{\rm cat}|, \rho(t)]$ are exhibited in Fig. S3(a). The success probability is defined by the fidelity, $\mathscr{F}[|\Psi_{\rm cat}\rangle\langle\Psi_{\rm cat}|, \rho(T_p)]$, corresponding to the time when the pump field is fixed.

Figure S3(b) shows the dependence of the success probability on T_p for various values of κ. The efficiency of the protocol is degraded by nonadiabatic transitions for small T_p and by photon loss for large T_p. The maximum success probability is lower than that of the measurement-based protocol for the stable coherent states. Although the success probability of creation of a cat state can be increased by tailoring the time dependence of detuning[6], such control is out of the scope of this paper aiming to propose a simple method.

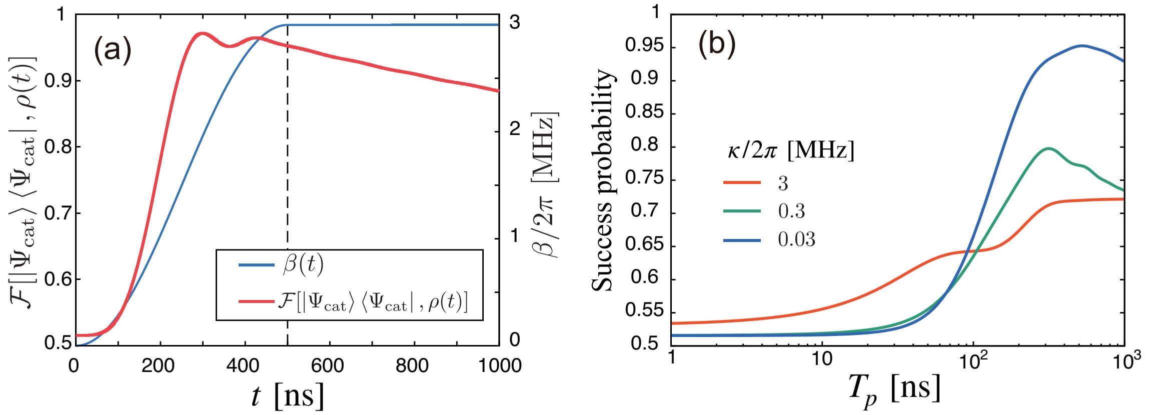

Figure S3. (a): Time dependence of β and the corresponding fidelity $\mathscr{F}[|\Psi_{\rm cat}\rangle\langle\Psi_{\rm cat}|, \rho(t)]$ for $T_p = 500$ ns and $\beta_0/2\pi = 3$ MHz. The dashed line indicates $t = T_p$. (b): T_p-dependence of the success probability defined by fidelity, $\mathscr{F}[|\Psi_{\rm cat}\rangle\langle\Psi_{\rm cat}|, \rho(T_p)]$ for various values of κ. The other parameters are the same as in Fig. 2.

.

References

1. Goto, H. Quantum computation based on quantum adiabatic bifurcations of Kerr-nonlinear parametric oscillators. *J. Phys. Soc. Jpn.* **88,** 061015 (2019).
2. Wang, Z., Pechal, M., Wollack, E. A., Arrangoiz-Arriola, P., Gao, M., Lee, N. R. & Safavi-Naeini, A. H. Quantum dynamics of a few-photon parametric oscillator. *Phys. Rev. X* **9,** 021049 (2019).
3. Puri, S. *et al.* Stabilized cat in a driven nonlinear cavity: a fault-tolerant error syndrome detector. *Phys. Rev. X* **9,** 041009 (2019).
4. Suzuki, S., Kawabata, S., Yamamoto, T. & Masuda, S. Quantum state tomography for Kerr parametric oscillators. arXiv:2212.14627 (2023).
5. Yamaji, T., Kagami, S., Yamaguchi, A., Satoh, T., Koshino, K., Goto, H., Lin, Z. R., Nakamura, Y. & Yamamoto, T. Spectroscopic observation of the crossover from a classical Duffing oscillator to a Kerr parametric oscillator. *Phys. Rev. A* **105,** 023519 (2022).
6. Masuda, S., Ishikawa, T., Matsuzaki, Y. & Kawabata, S. Controls of a superconducting quantum parametron under a strong pump field. *Sci. Rep.* **11,** 11459 (2021).
